# Tapia's Syndrome after Corrective Jaw Surgery under General Anesthesia: A Case Report

**Published:** 2017-03

**Authors:** Farzad Izadi, Aslan Ahmadi, Ali Daneshvar, Mahdi Safdarian

**Affiliations:** 1 *ENT-Head and Neck Surgery Research Center, Hazrat Rasoul Akram Hospital , Iran University of Medical Science, Tehran, Iran*

**Keywords:** Airway Management, General Anesthesia, Vocal Cord paralysis

## Abstract

**Introduction:**

Tapia’s syndrome is a rare complication of recurrent laryngeal and hypoglossal nerve paralysis due to anesthetic airway mismanagement or malpositioning of the patient’s head during surgery.

**Case Report:**

Here we present a case of Tapia's syndrome in a 22-year-old male after corrective jaw surgery under general anesthesia, with a long period of recovery, related to airway management procedures and/or overstretching of the neck during positioning for surgery.

**Conclusion:**

Although it is a rare condition, every surgeon should be aware of Tapia’s syndrome in order to consider the correct positioning of the head and endotracheal tube during surgery and avoid this complication.

## Introduction

Tapia’s syndrome, first described by Tapia in 1904, is a rare complication of extracranial involvement of the laryngeal and hypoglossal nerves, resulting in the recurrent ipsilateral paralysis of the vocal cord and tongue (1). Tapia’s syndrome is usually a complication related to anesthetic airway management and positioning of the patient’s head during surgery, and the mechanism of injury is believed to be neurapraxic due to pressure neuropathy (2). The most common clinical symptoms are hoarseness, difficulty in tongue movement, dysphagia, and even dyspnea (3). Orthognathic or corrective jaw surgery involves procedures on the upper jaw (maxilla), lower jaw (mandible), cheekbones, and nasal bones as well as other facial bones.

We describe here a case of Tapia’s syndrome following corrective jaw surgery. The patient has consented to submission of the case report for publication.

## Case Report

**Fig 1:**
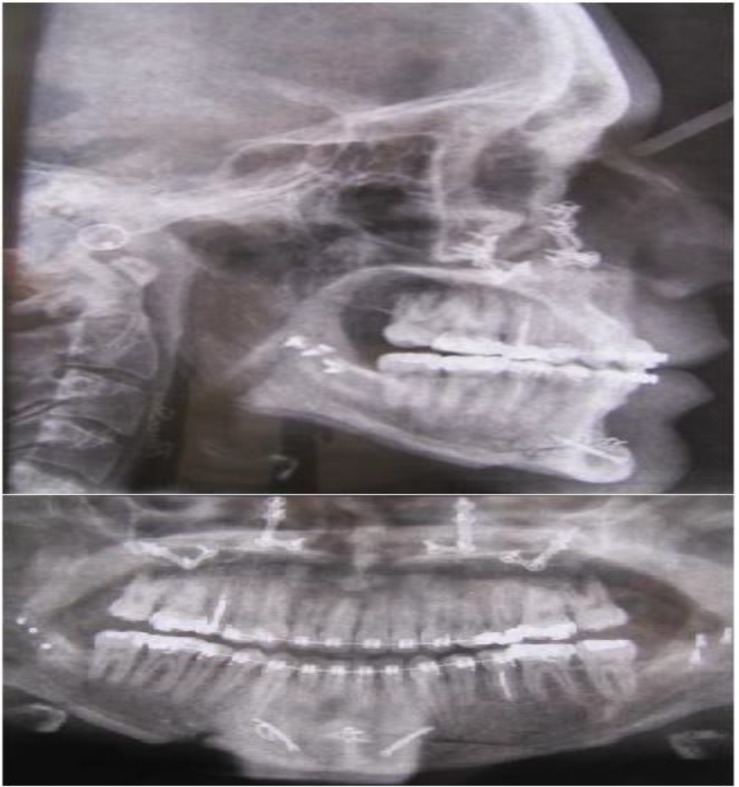
Corrective jaw surgery with 24 screws in jawbones

A 22-year-old man (61kg) went under corrective jaw surgery because of the deviation of two teeth; one in the maxilla and the other in mandible (Fig.1).

**Fig 2:**
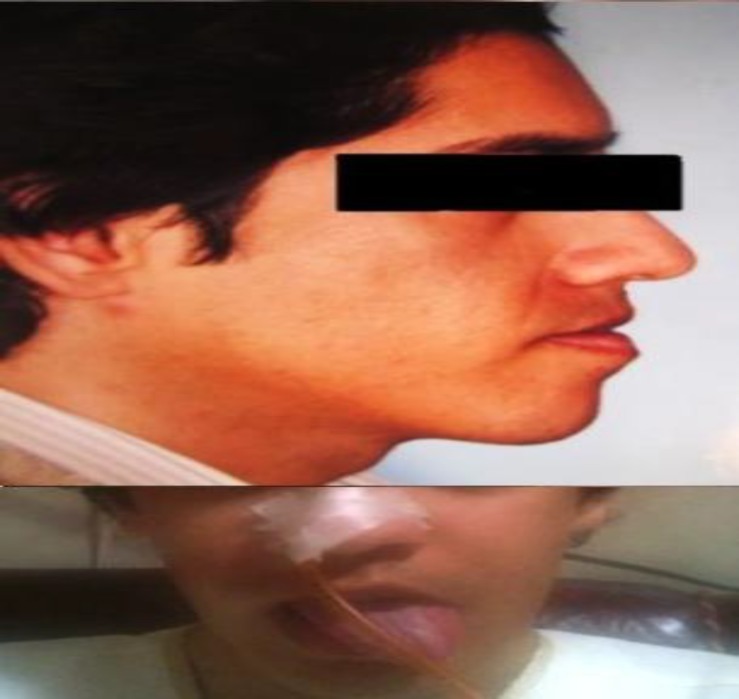
The patient before (top) and 1 month after (bottom) the corrective jaw surgery. Tongue deviation due to hypoglossal nerve paralysis is seen

 Despite positioning of the upper and lower teeth over each other, the jaws were not completely locked with the lower jaw being placed slightly forward. One day after surgery, the patient complained of dysphonia, hoarseness of voice, inability to swallow, and tongue palsy (Fig.2). 

An ear, nose, and throat (ENT) and neurology examination confirmed deviation of the tongue and left vocal cord paralysis. There was no edema or hematoma in the pharynx or larynx. A computerized tomography scan was reported as normal without any abnormality. Magnetic resonance imaging was not performed due to the inability of the patient to sustain the supine position. Other differential diagnoses were excluded by imaging and accurate history taking. The patient had no comorbidities before surgery that could justify the symptoms.

The patient received nutrition through a percutaneous endoscopic gastrostomy (PEG) and remained under suctioning for about 2 months after surgery. He made a gradual recovery over the next few weeks, and the PEG was removed.

The left vocal cord was still paralyzed 6 months after surgery. Four months later, the patient gradually began to speak. At a10-month follow-up visit, the patient had completely recovered and the syndrome resolved with no neurological deficit.

A clinical diagnosis of Tapia’s syndrome was made by the first author 12 months after the first corrective surgery.

## Discussion

The most likely mechanism of Tapia’s syndrome, associated with general anesthesia, includes direct compression of the recurrent laryngeal and hypoglossal nerves by an endotracheal tube and a prolonged stretch of these nerves following excessive flexion of the head (4). In the literature review by Gevorgyan and Nedzelski, the mechanism of Tapia's syndrome was reported to be associated with airway management in 70% of patients due to the compression or stretching of recurrent laryngeal and hypoglossal nerves during surgery. This review reported excellent recovery in 30% of patients, incomplete recovery in 39%, and no recovery in over 26% of patients (5). Since the mechanism of injury is believed to be neurapraxic, recovery largely depends on time and supportive treatment, with an emphasis on empiric corticosteroids and dysphagia therapy. In our case, for example, total recovery was achieved approximately 10 months after the first surgery.

This complication can occur in any surgery under general anesthesia. For example, Park et al. reported a case of Tapia’s syndrome after posterior cervical spine surgery (6), while Lim et al. reported a case of this syndrome following cervical laminoplasty (2).

Many studies have reported this complication as a result of airway management with an endotracheal tube; however some cases have been reported after other procedures such as cardiac surgery (3), coronary bypass grafting surgery (7), and rhinoplasty (8). Ghorbani et al. reported a rare case of co-presentation of Tapia’s syndrome and pressure alopecia after septorhinoplasty (9). Further, there was one case of this syndrome reported after repair of a fractured mandible, by Kashyap et al. (10). From our search of the literature, we believe this is the first case of Tapia's syndrome reported following orthognathic surgery.

## Conclusion

Although Tapia’s syndrome is a rare complication, it is necessary that both surgeons and anesthesiologists are aware of this condition. We recommend careful attention to the correct positioning of the head during surgery and airway management in order to avoid this complication.
